# Prognostic value of tumor-infiltrating B lymphocytes and plasma cells in triple-negative breast cancer

**DOI:** 10.1007/s12282-021-01227-y

**Published:** 2021-02-25

**Authors:** Hajime Kuroda, Tsengelmaa Jamiyan, Rin Yamaguchi, Akinari Kakumoto, Akihito Abe, Oi Harada, Bayarmaa Enkhbat, Atsuko Masunaga

**Affiliations:** 1 Department of Diagnostic Pathology, Medical Center East, Tokyo Women’s Medical University, 2-1-10 Nishiogu, Arakawa-ku, Tokyo, 116-8567 Japan; 2 2Department of Diagnostic Pathology, Dokkyo Medical University, Mibu, Japan; 3 3Department of Pathology and Forensic Medicine, Mongolian National University of Medical Sciences, Ulan Bator, Mongolia; 4 4Department of Pathology & Laboratory Medicine, Kurume University Medical Center, Kurume, Japan; 5 5Department of Diagnostic Pathology, Nasu Red Cross Hospital, Otawara, Japan; 6 Breast Center, Dokkyo Medical University, Mibu, Japan; 7 Breast Center, Showa University, Tokyo, Japan

**Keywords:** Breast, Triple negative cancer, Prognostic factor, CD20, CD38, CD138

## Abstract

**Background:**

Recent investigations have demonstrated that the tumor microenvironment, including tumor-infiltrating lymphocytes (TILs), is an important factor in tumor growth and development. While the prognostic correlation of tumor-infiltrating T cells has been widely studied in breast cancer, that of tumor-infiltrating B cells and plasma cells has not received so much attention, especially in triple-negative breast cancer (TNBC).

**Methods:**

We investigated 114 patients with TNBC who had surgery between 2006 and 2019 at Dokkyo Medical University Hospital. Intratumoral (i) TILs were considered to be lymphocytes within cancer cell nests and directly infiltrating tumor cells. Similarly, stromal (s) TILs were considered to be lymphocytes within the tumor stroma, but not directly infiltrating tumor cells. CD20 + , CD38 + and CD138 + staining was determined by estimating the number of positive B cells.

**Results:**

sCD20 + TILs had prognostic significance for relapse-free survival (RFS) (*p* = 0.043) and overall survival (OS) (*p* = 0.027). The sCD38 + TILs were significantly related to favorable RFS (*p* = 0.042). iCD38, iCD138, and sCD138 was not significantly correlated with RFS (*p* = 0.065, *p* = 0.719, *p* = 0.074) or OS (*p* = 0.071, *p* = 0.689, *p* = 0.082).

**Conclusions:**

The present study demonstrated that a high density of sCD20 + TILs was significantly related to favorable prognosis in both RFS and OS. Increased sCD38 + TILs in TNBC were correlated with a significantly favorable prognosis in RFS. These results indicate that TILs–B may have a profound influence on the clinical outcome of TNBC.

## Introduction

Recent investigations have demonstrated that the tumor microenvironment, including tumor- infiltrating lymphocytes (TILs), is an important factor in tumor growth and development. While the prognostic correlation of tumor-infiltrating T cells has been widely studied in breast cancer, that of tumor-infiltrating B cells (TILs – B) and plasma cells has not received so much attention, especially in triple-negative breast cancer (TNBC). Several studies immunohistochemically investigated the prognostic influence of CD20 + , CD38 + , and CD138 + TILs on esophageal cancer, gastric cancer, colorectal cancer, melanoma, ovarian cancer, and non-small cell lung cancer [1–6]. Recently, several breast cancer researchers have reported that a predominance of TILs – B was correlated with favorable prognosis [7–13], while other studies discovered a negative association with prognosis [14, 15]. The potential mechanisms of these results can be explained by B-cell characteristics; they may participate in the secretion of effector cytokines that can polarize T cells towards a Th1 or Th2 reaction, activate T-cell reactions through their role as antigen-presenting cells, while direct cytotoxicity in cancer has also been suggested [16]. Tertiary lymphoid structures (TLS) are constructed aggregates of lymphoid cells that resemble secondary lymphoid organs [17]. TLS present in human solid tumors are important to construct a favorable immune microenvironment to control tumor progression [17, 18]. Their functions include B-cell activation, differentiation into plasma cells and antibody production [19] associated with anti-tumor responses [17, 19]. Invasive breast cancer has been categorized into several major subtypes based on gene expression profiles [20]. The term TNBC is defined by loss of the estrogen receptor (ER) and progesterone receptor (PgR), and human epidermal growth factor receptor 2 (HER2) protein overexpression, which is known to have a negative prognosis.

The correlation between TILs-B such as CD20 + , CD38 + , and CD138 + , TLS, and breast cancer, especially in TNBC, is still being debated. We herein discuss the clinicopathological features and prognostic value of TILs-B analyzed with CD20 + , CD38 + , and CD138 + in TNBC.

## Materials and methods

### Patients

We investigated 114 patients with TNBC who had surgery between 2006 and 2019 at Dokkyo Medical University Hospital. Hematoxylin and eosin (H&E) stained whole-tissue sections were estimated by two investigators (HK and TJ) without clinical information or prior histological results. Ethical approval for this study was obtained from the Dokkyo Medical University Ethics Committee.

### Immunohistochemistry (IHC)

Using IHC, the specimens were stained with the following antibodies: ER (clone SP1, Novocastra (Leica), prediluted, nuclear), PgR (clone 1E2, Novocastra (Leica), prediluted, nuclear), HER2 (clone 4B5, Roche (VENTANA), prediluted, membranous), CD20 (CD20, clone L26, nichirei), CD38 (CD38, clone SPC32, Novocastra (Leica) 1:200), and CD138 (CD138, clone MI15, Novocastra (Leica)). Hematoxylin was used for counterstaining. The percentages of ER and PgR were estimated, as mentioned by the guideline, and a patient was determined to be “positive” if the breast tumor was confirmed to have at least 1% positive cells [21]. HER2-negative was considered to be staining with a score of 0/1 + . For score 2 staining, fluorescence in situ hybridization (FISH) was considered positive for HER2 amplification when the ratio of HER2 to chromosome enumeration probe 17 (CEP17) was > 2.0 [22]. For mib-1 expression, the cut-point value was set at 20% based on a previous report [23].

**Fig. 1:**
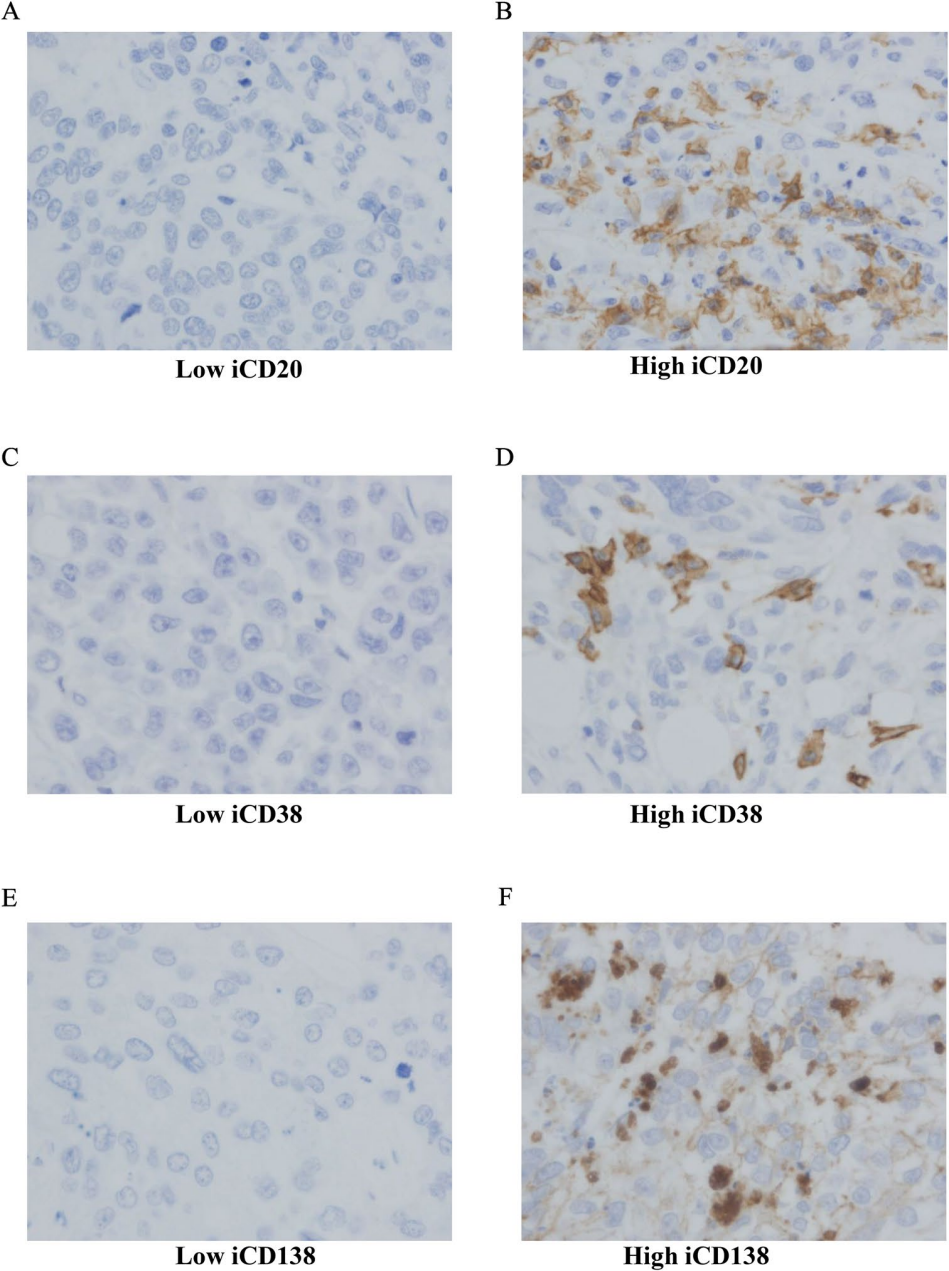
Immunohistochemical detection of intratumoral (i) TILs (CD20 + , CD38 + , and CD138 +) in triple-negative breast cancer (TNBC). **a** and **b** Low vs high expression of iCD20 + TILs in patients’ specimens. **c** and **d** Low vs high expression of iCD38 + TILs in patients’ specimens. **e** and **f** Low vs high expression of iCD138 + TILs in TNBC. (original magnification × 400)

**Fig. 2:**
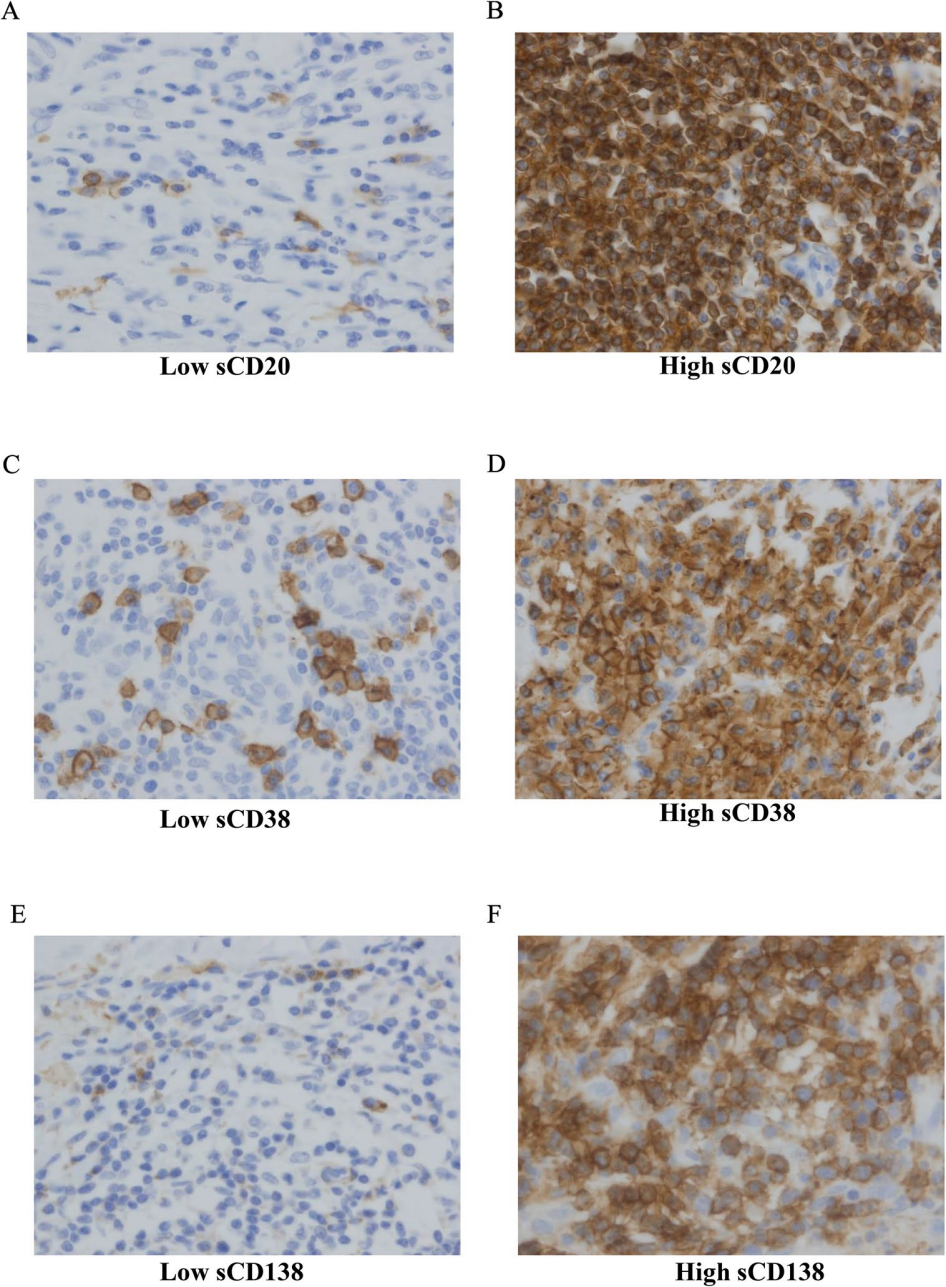
Stromal(s) TILs (CD20 + , CD38 + , and CD138 +) in triple-negative breast cancer (TNBC). Immunohistochemical staining representing low (**a**) and high (**b**) sCD20 + TILs density; low (**c**) and (**d**) high sCD38 + TILs density; and low (**e**) and high (**f**) sCD138 + TILs density in TNBC. (original magnification × 400)

Assessment of the TILs on H&E-stained full-face sections was conducted according to the International Immuno-Oncology Biomarkers Working Group [24]. We evaluated two locations of TILs, the intratumoral (i) and stromal (s) parts, individually. iTILs were assessed as lymphocytes in direct cell-to-cell contact with tumor cells with no intervening stroma. sTILs were assessed as lymphocytes within the tumor stroma and not directly infiltrating tumor cells. The cut-point value was set at 30% [25]. TLS were evaluated on whole H&E sections and TLS-positive tumors were defined as having ≥ 1 TLS, and TLS-negative tumors were defined by the absence of TLS (< 1). The CD20 + , CD38 + , and CD138 + TILs were determined by calculating the number of positive cells [26]. For statistical analyses, the number of positive cells was categorized into low and high groups according to the cut-point value using the median (Figs. 1, 2).

### Statistical analysis

The correlations between CD20 + , CD38 + , and CD138 + TILs and clinicopathological factors were assessed by the *x*^2^*-*test. Relapse–free survival (RFS) was determined as the date of surgery to recurrence including loco-regional, or distant metastasis. Overall survival (OS) was defined as the time from the primary surgery to death by cancer or the time to the last follow-up for patients still alive. The RFS and OS curves were assessed using the Kaplan–Meier method, and the results were compared using the log-rank test. Univariate and multivariate Cox proportional hazard models were applied to identify clinicopathological factors that were correlated with survival rate, and hazard ratios (HRs) were noted as point evaluations with 95% confidence intervals (CI). IBM SPSS Statistics 26 (IBM, Armonk, NY, United States) was used for statistical analyses. Data were assumed to be statistically significant at *p* < 0.05, and all *p* values were two-tailed.

## Results

The median age of breast cancer patients was 62 years (range 28–89) and 59 (51.8%) were older than 60 years. The majority had an invasive ductal carcinoma (IDC) 88 (77.2%), and a high histological grade 79 (69.3%). There were 71 tumors of ≤ 2 cm (62.3%) and high mib-1 was observed in 93 (81.6%) patients. Recurrence occurred in 22 (19.3%), and cancer-correlated death occurred in 21 (18.4%), of 114 patients. Median follow-up for the RFS analysis was 35 months and that for OS was 42.5 months. The cut-point value for iCD20 + was 1, sCD20 + was 61, iCD38 + was 4, sCD38 + was 39.5, iCD138 + was 10.5, and sCD138 + was 35. The clinicopathological characteristics of the 114 TNBC patients in terms of i and s CD20 + TILs, CD38 + TILs and CD138 + TILs are presented in Table 1. A low density of iCD20 + TILs was significantly correlated with a lower density of iTILs (*p* = 0.002) and sTILs (*p* = 0.004). However, there was no significant association between a predominance of iCD38 + TILs and iCD138 + TILs and clinicopathological characteristics. A predominance of sCD20 + TILs, sCD38 + TILs, and sCD138 + TILs was associated with higher sTILs expression (*p* < 0.001, *p* < 0.001, and *p* = 0.011, respectively). A predominance of sCD20 + TILs showed a significant difference for iTILs (*p* = 0.009), while sCD138 + TILs were associated with mib-1 (*p* = 0.032) expression. In addition, the presence of TLS was significantly associated with high iCD20, iCD138, sC20, and sCD138 (*p* = 0.008, *p* = 0.023, *p* < 0.001, and *p* = 0.036).

**Table 1:** Clinicopathological features of triple-negative breast cancer (TNBC) and the status of intratumoral and stromal CD20, CD38, and CD138 (*N*= 114)

Clinicopathological feature	Total no. of cases	iCD20			iCD38			iCD138			sCD20			sCD38			sCD138		
		Low	High	*P* value*	Low	High	*P* value*	Low	High	*P* value*	Low	High	*P* value*	Low	High	*P* value*	Low	High	*P* value
Age (years)				0.209			0.053			0.349			0.574			0.574			0.432
<60	55	32	23		36	19		25	30		26	29		26	29		23	32	
≥60	59	41	18		28	31		32	27		31	28		31	28		29	30	
Tumor size (cm)				0.536			0.221			0.562			0.176			0.344			0.311
≤2	71	47	24		43	28		34	37		32	39		38	33		35	36	
>2	43	26	17		21	22		23	20		25	18		19	24		17	26	
Histological grade				0.052			0.580			0.155			0.542			0.310			0.100
I and II	35	27	8		21	14		14	21		19	16		20	15		20	15	
III	79	46	33		43	36		43	36		38	41		37	42		32	47	
Histology				0.132			0.111			0.258			0.899			0.258			0.992
IDC	88	56	32		45	43		45	43		13	45		43	45		40	48	
ILC	2	0	2		1	1		2	0		1	1		0	2		1	1	
Other types	24	17	7		18	6		10	14		13	11		14	10		11	13	
Mib-1				0.199			0.918			0.469			0.469			0.227			0.032*
<20%	21	16	5		12	9		9	12		12	9		13	8		14	7	
≥20%	93	57	36		52	41		48	45		45	48		44	49		38	55	
iTILs				0.002*			0.868			0.454			0.009*			0.143			0.561
Low	58	45	13		33	25		31	27		36	22		33	25		28	30	
High	56	28	28		31	25		26	30		21	35		24	32		24	32	
sTILs				0.004*			0.406			1.000			<0.001*			<0.001*			0.011*
Low	62	47	15		37	25		31	31		47	15		44	18		35	27	
High	52	26	26		27	25		26	26		10	42		13	39		17	35	
TLS				0.008*			0.540			0.023*			<0.001*			0.148			0.036*
Absent	81	58	23		44	37		46	35		51	30		44	37		42	39	
Present	33	15	18		20	13		11	22		6	27		13	20		10	23	

*TNBC* triple-negative breast cancer, *IDC* invasive ductal carcinoma, *ILC* invasive lobular carcinoma, *TLS* tertiary lymphoid structures, *i* intratumoral, *s* stromal, *TILs* Tumor-infiltrating lymphocytes

*p value is significant, *χ*^2^ test

Correlations among prognosis and clinicopathological factors, unstained TILs, CD20 + TILs, CD38 + TILs, and CD138 + TILs are listed in Table 2. Multivariate Cox regression analysis was applied for all clinicopathological factors that were significantly correlated with survival in univariate analysis for RFS and OS. The results revealed that larger tumor size was related to poorer RFS (HR = 2.616, 95% CI 1.082–6.330, *p* = 0.033) and OS (HR = 2.849, 95% CI 1.166–6.962, *p* = 0.022). sCD20 + TILs was significant for RFS with HRs of 0.363 (95% CI 0.136–0.969, *p* = 0.043) and OS with HRs of 0.330 (95% CI 0.124–0.880, *p* = 0.027). sCD38 + TILs were significantly correlated with favorable RFS (HR = 0.323, 95% CI 0.109–0.960, *p* = 0.042), but not OS (HR = 0.342, 95% CI 0.113–1.037, *p* = 0.058).

**Table 2:** Relapse-free survival (RFS) and overall survival (OS) rate according to Univariate and Multivariate Cox regression analyses of prognostic factors of 114 patients with triple-negative breast cancer (TNBC)

Clinicopathological feature	RFS				OS			
	Univariate analysis		Multivariate analysis		Univariate analysis		Multivariate analysis	
	HR (95.0% CI)	*P* value*	HR (95.0% CI)	*P* value*	HR (95.0% CI)	*P* value*	HR (95.0% CI)	*P* value*
Age (<60 vs. ≥60)	0.523 (0.209–1.308)	0.166			0.564(0.225–1.413)	0.222		
Tumor size (2 cm vs. >2 cm)	2.491(1.051–5.905)	0.038*	2.616(1.082–6.330)	0.033*	2.710(1.139–6.452)	0.024*	2.849(1.166–6.962)	0.022*
Histological grade (I, II vs. III)	2.666(0.785–9.055)	0.116			2.582(0.760–8.770)	0.128		
Histology (IDC vs. ILC, other types)	0.590(0.173–2.007)	0.398			0.600(0.176–2.040)	0.413		
Mib-1 (<20% vs. ≥20%)	2.176(0.500–9.464)	0.300			2.379(0.542–10.448)	0.251		
TLS (absent vs. present)	0.776(0.283–2.124)	0.621			0.772(0.283–2.112)	0.615		
iTILs (low vs. high)	0.598(0.244–1.468)	0.262			0.565(0.230–1.390)	0.214		
sTILs (low vs. high)	0.330(0.111–0.988)	0.048*	0.563(0.169–1.877)	*0.349*	0.332(0.111–0.992)	0.048*	0.569(0.168–1.933)	*0.366*
iCD20 (low vs. high)	0.273(0.080–0.928)	0.037*	0.373(0.106–1.310)	*0.124*	0.273(0.080–0.929)	0.038*	0.377(0.108–1.320)	*0.127*
sCD20 (low vs. high)	0.287(0.110–0.746)	0.010*	0.363(0.136–0.969)	0.043*	0.265(0.102–0.690)	0.007*	0.330(0.124–0.880)	0.027*
iCD38 (low vs. high)	0.388(0.142–1.061)	0.065			0.396(0.145–1.083)	0.071		
sCD38 (low vs. high)	0.280(0.102–0.764)	0.013*	0.323(0.109–0.960)	0.042*	0.292(0.107–0.799)	0.017*	0.342(0.113–1.037)	*0.058*
iCD138 (low vs. high)	1.173(0.492–2.794)	0.719			1.193(0.502–2.838)	0.689		
sCD138 (low vs. high)	0.446(0.184–1.082)	0.074			0.455(0.187–1.105)	0.082		

*TNBC* triple-negative breast cancer, *RFS* relapse-free survival, *OS* overall survival, *HR* hazard ratio, *CI* confidence interval, *IDC* invasive ductal carcinoma, *ILC* invasive lobular carcinoma, *TLS* tertiary lymphoid structures, *i* intratumoral, *s* stromal, *TILs* tumor-infiltrating lymphocytes

*p value is significant

**Fig. 3:**
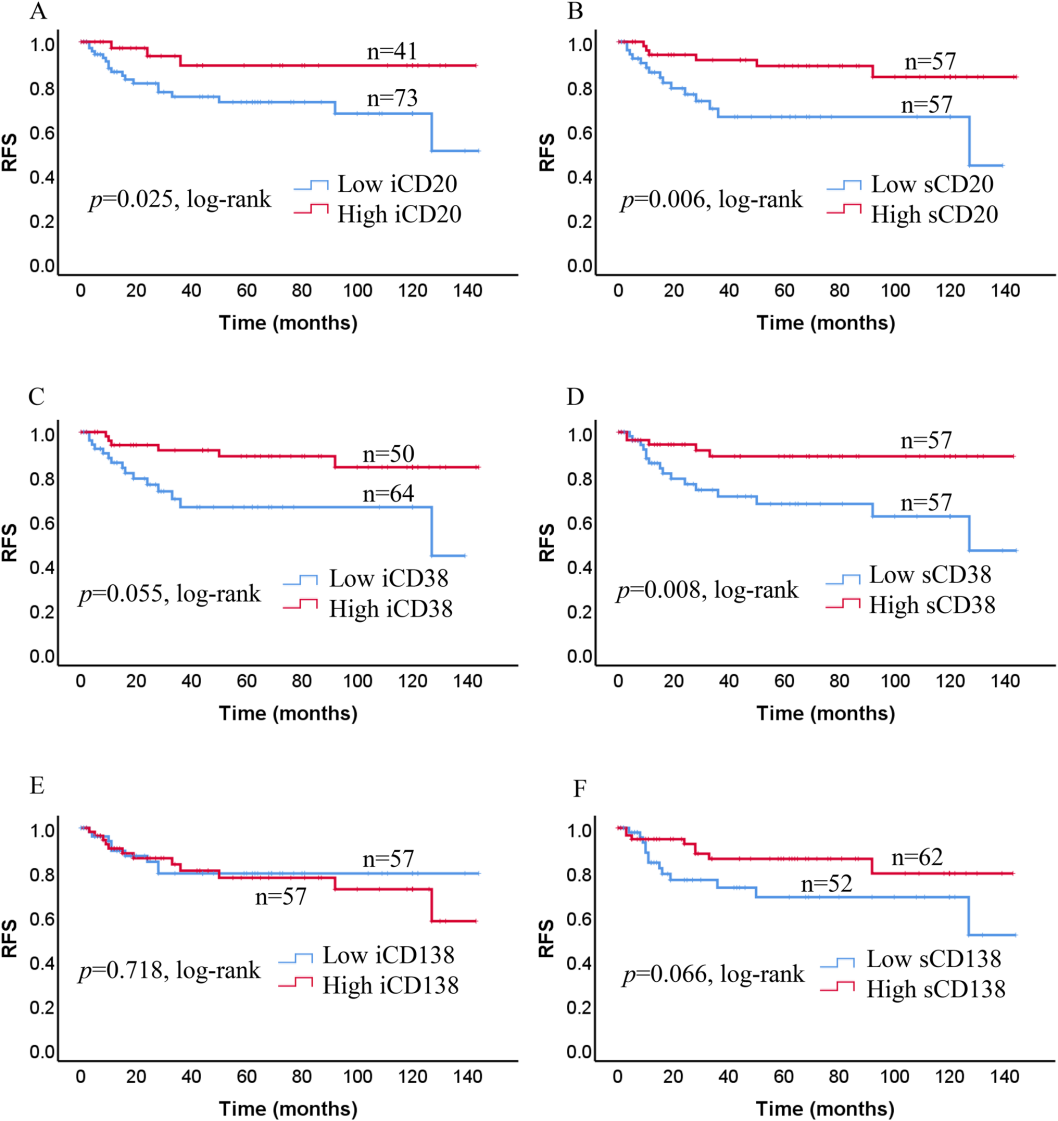
Kaplan–Meier estimates of relapse-free survival (RFS) in all patients based on i and s TILs (CD20 + , CD38 + and CD138 +) expression. Kaplan–Meier analysis of RFS in low and high expression curves of **a** iCD20 + TILs, **b** sCD20 + TILs, **c** iCD38 + TILs, **d** sCD38 + TILs, **e** iCD138 + TILs, and **f** sCD138 + TILs

**Fig. 4:**
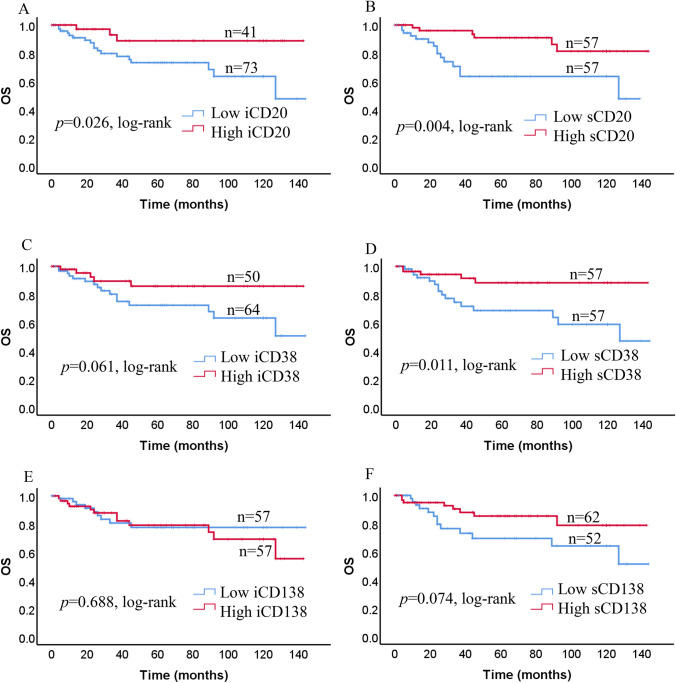
Kaplan–Meier curves of overall survival (OS) in triple-negative breast cancer (TNBC). OS based on high and low expression of iCD20 + TILs (**a**), sCD20 + TILs (**b**), iCD38 + TILs (**c**), sCD38 + TILs (**d**), iCD138 + TILs (**e**), and sCD138 + TILs (**f**) in all patients with TNBC

We analyzed survival in terms of the significant rates of CD20 + TILs, CD38 + TILs, and CD138 + TILs using the Kaplan–Meier method and log-rank test. TNBCs classified as high iCD20 + TILs, sCD20 + TILs, and sCD38 + TILs had significantly improved RFS compared to those with low expressions of iCD20 + TILs, sCD20 + TILs, and sCD38 + TILs (*p* = 0.025, *p* = 0.006, *p* = 0.008, respectively; Fig. 3a, b, d). Nevertheless, no significant association was found between the density of iCD38 + TILs (*p* = 0.055), iCD138 + TILs (*p* = 0.718), and sCD138 + TILs (*p* = 0.066) and RFS (Fig. 3c, e, f). A high density of iCD20 + TILs, sCD20 + TILs and sCD38 + TILs was significantly related to better OS compared with a low density of iCD20 + TILs, sCD20 + TILs, and sCD38 + TILs (*p* = 0.026, *p* = 0.004, and *p* = 0.011, respectively; Fig. 4a, b, d). In contrast, no correlation between the status of iCD38 + TILs, iCD138 + TILs, and sCD138 + TILs and OS was observed (*p* = 0.061, *p* = 0.688, and *p* = 0.074, respectively; Fig. 4c, e, f).

## Discussion

We found that TNBC patients with a significant increase in sCD20 + TILs had a favorable prognosis in terms of both RFS and OS. Previous studies provided evidence that significant expression of TILs–B was correlated with favorable prognosis in several organs [1, 2, 6, 27–29]. A high distribution of CD20 + TILs in epithelioid mesothelioma was correlated with good prognosis [30]. Further, CD20 + TILs had positive associations in high grade serous ovarian cancer [5], but not in other histological subtypes [31]. Similar results have been confirmed in several organs such as ovarian, gastric, and colorectal cancers [3, 32, 33]. Therefore, it is possible to predict that in cancers of certain organs, expression of an immune suppressive substance from TIL–B supports anti-tumor cells. However, the limited data on the role of TIL–B in breast cancer is still being debated. Mohammed et al. reported that the proportion of CD20 + TILs did not correlate with outcomes in primary breast cancer [15]. In contrast, previous studies reported on the role of CD20 + TILs in breast cancer and found a positive prognostic effect [7, 8, 10, 11, 34]. In multivariable analysis, Brown et al. reported that only CD20 + TILs in breast cancer were associated with a significant complete pathologic response (pCR) [35]. Moreover, these results were independent of other variables such as age, tumor size, nuclear grade, lymph node status, ER, PgR, HER2 status and ki67 index. Song et al. also reported that a high density of CD20 + TILs was a significant predictor of pCR in a total of 108 TNBC patients treated with neoadjuvant chemotherapy [9]. Xu et al. recently reported in a multivariate analysis of 102 IDC patients that a high level of CD20 + TILs showed improved OS, but not good disease-free survival (DFS) [12]. Furthermore, in an extensive number of invasive breast cancers, Mahmoud et al. observed predominance of CD20 + TILs was significantly correlated with favorable prognosis in both breast cancer-specific survival and disease-free interval [8]. However, these two reports used tissue microarrays that can underestimate TIL–B compared with full sections. The present study evaluated whole sections from each case for which iCD20 + and sCD20 + TILs correlated with good prognosis and TILs–B assessed by CD20 + indicated promotion of an immune reaction; therefore, CD20 + cells likely have an essential role in the tumor progression associated with TNBC prognosis.

In univariate analyses, we found that TNBC patients with a favorable prognosis in terms of both RFS and OS had a high density of sCD38 + TILs, but not CD138 + TILs. However, in multivariate analyses, we only observed that TNBC patients with an improved prognosis had an increased number of sCD38 + TILs in RFS. Only a few studies reported on the role of CD138 + TILs in cancers and they were correlated with negative prognosis.　Lundgren et al. reported that a high density of CD20 + and CD138 + TILs was markedly associated with high grade ovarian cancer [36]. Univariate Cox regression analysis indicated that a high density of CD138 + TILs was correlated with poor disease-specific survival and OS. Similar to these findings, Bosisio et al. reported that melanomas with a high number of CD138 + TILs were associated with worse prognosis [4]. In contrast to our findings, Mohammed et al. reported that CD138 + TILs were negatively associated with RFS in ductal breast cancer in multivariate analysis [15]. Milligy et al. reported that a high density of CD138 + TILs among TILs in patients correlated with shorter RFS [14]. However, several studies showed an improved prognostic influence of CD138 + TILs in melanoma, esophageal cancer, gastric cancer, and colorectal cancer [1, 3, 28, 37]. As the results differ among these reports, it is difficult to evaluate CD138 + . In our study, CD138 + stained not only TILs–B, but also epithelial and stromal cells. Due to estimation difficulty, there was no significant difference in iCD138 + and sCD138 + TILs in our study. Tumor epithelium and stromal CD138 + reactions in breast cancer have been reported previously [34, 38]. Therefore, it is not unexpected that different prognostic results have been reported for CD138 + . Further, similar to CD138 + , there are reports of CD38 + TILs, a marker of not only plasma cells, but also defining naïve memory B cells, in several solid tumors. The elucidating role of the CD38 marker may be associated with the development and immune escape within solid tumors and is a relatively new concept. The limited data in melanoma, glioma, esophageal, cervical, and lung cancers indicate an immunosuppressive role for CD38 [6, 39–43]. In these solid tumors, CD38 seems to be activated as a tumor-progressing factor. However, the role of CD38 in solid tumors is likely to be more complex than first considered. Recently, CD38 + TILs have been associated with longer survival in urinary bladder cancer [44]. Similar to our findings from whole section examinations, Yeong et al. recently used the tissue microarray method and multivariate analysis to clarify that TNBC patients with CD38 + TILs had significantly better DFS, but not OS [34]. Therefore, these results also imply that increased proliferation of CD38 + may have an effect on the clinical outcome in TNBC.

CD38 was primarily reported as a lymphocyte marker [45, 46], but knowledge about CD38 has advanced [47, 48]. It is almost ubiquitously expressed on multiple immune cells, including not only B cells, but also T cells, NK cells, macrophages and dendritic cells [49, 50]. These studies have shown the detection of CD38 + on IHC-stained tissue alone, leads to under or overestimation. Therefore, we observed CD38 + B cells on both H&E slides and IHC-stained slides to generate greater intra- and inter-observer agreement and reproducibility. However, it is possible that we may not have completely excluded CD38 expression on cells within the tumor microenvironment, such as T cells, macrophages and dendritic cells. While CD38 expression on B cells remains the main theme of current research, more data is beginning to be gathered concerning the role of CD38 expression on tumor cells. Additional research is expected to completely elucidate its function and potential in TNBC.

TLS are highly constructed aggregates of lymphoid cells that resemble lymph nodes [18]. It has been suggested that TLS support anti-tumor reactions involving the combined responses of both B-cell activation and antibody-production by plasma cells [17]. Previous studies reported the close correlation of TLS in tumors of the ovary [18], breast [51, 52], metastatic melanoma [28], and non-small cell lung cancer [53]. Further, Lee et al. reported that TLS is an important factor in the pathological complete response in TNBC [51]. Further, Seow et al. reported that TNBC with a high density of CD20 + and CD38 + B cells were associated with TLS [54]. However, tumor areas were selected for tissue microarray (TMA) construction in their research. The limitation of TLS detection on TMA alone can lead to an incorrect evaluation, as compared to detection of TLS on whole slide sections. Alhough only certified in a limited cohort, our findings also suggest that higher densities of CD20 and CD138 + cells were also associated with TLS in TNBC. However, evidence for this was not confirmed by multivariate analysis. More research using a larger cohort should be performed, not only to establish the proportion and frequencies of TLS in TNBC, but also to clarify the association between TLS and B cells in forming an anti-tumoral microenvironment for TNBC.

## Conclusions

The present study demonstrated that a higher density of sCD20 + TILs was significantly related to favorable prognosis in both RFS and OS. Increased sCD38 + TILs in TNBC were correlated with a significantly favorable prognosis in RFS. These results indicate that TILs–B may have an improved prognostic influence on clinical outcomes in TNBC.
